# El futuro de la salud pública tras la pandemia. Una ventana de oportunidad

**DOI:** 10.23938/ASSN.1045

**Published:** 2023-08-22

**Authors:** Salvador Peiró

**Affiliations:** Área de Investigación en Servicios de Salud y Farmacoepidemiología Fundación para el Fomento de la Investigación Sanitaria y Biomédica de la Comunidad Valenciana (FISABIO) València España

El trabajo de Casado y col^1^ que publica Anales del Sistema Sanitario de Navarra en este número describe, en sus cifras principales de infecciones, hospitalizaciones, ingresos en críticos y defunciones, el impacto de la pandemia en Navarra. No es lo único que sucedió. Hubo confinamientos, desempleo, empobrecimiento, problemas de salud mental, Covid-persistente, secuelas, etc. En otros trabajos del excelente monográfico de Anales dedicado al análisis de la pandemia en Navarra^2^ se abordan muchos de estos aspectos. Pero las cifras reportadas por Casado y colegas son demoledoras^1^. Suficientes para ilustrar el rigor con que la COVID-19 sacudió nuestras sociedades.

La COVID-19 ha mostrado, con toda crudeza, cómo la seguridad, la salud y la prosperidad económica de un país dependen de su capacidad para impedir (o minimizar) los efectos de algunas amenazas para la salud potencialmente devastadoras. Esa capacidad protectora está construida sobre la suficiencia, aptitud y talento de su sistema de salud pública, entendido como “*los esfuerzos organizados y las elecciones informadas de la sociedad, las organizaciones públicas y privadas, las comunidades y los individuos para prevenir enfermedades, prolongar la vida y promover la salud*”^3^.

Tradicionalmente invisible, la pandemia ha puesto de relieve este papel crítico de la salud pública abriendo una ventana de oportunidad para abordar su mejora y reenfocar su arquitectura y muchos de sus componentes. Una ventana para mejorar nuestra capacidad de abordar riesgos históricos y emergentes. Incluso en ausencia de *emergencias*, un sistema de salud pública robusto es imprescindible para proteger y mejorar la salud, prevenir la enfermedad y reducir las desigualdades. Más allá de virus y enfermedades transmisibles, la pandemia ha hecho emerger una problemática que conecta salud pública, control de zoonosis, seguridad alimentaria, resistencias bacterianas, cambio climático, calidad del aire y desigualdades sociales en un contexto de envejecimiento poblacional e incremento de la cronicidad.

Una oportunidad^4^. El futuro no está escrito en piedra. Pero una oportunidad de abandonar el prolongado descuido que ha ido reduciendo las capacidades de respuesta de la salud pública en España es más de lo que se entreveía hace unos años, mientras dormitaba sin desarrollo la Ley de Salud Pública de 2011^5^. Es una oportunidad para que los sistemas de salud pública (del Estado, las comunidades autónomas, las corporaciones locales) dispongan de una financiación suficiente, previsible, flexible y transparente para abordar la provisión de servicios de protección, promoción y prevención, las actuaciones sobre los determinantes sociales de salud y los asociados al envejecimiento y la cronicidad. También los problemas emergentes: la violencia, incluyendo la de género, las *nuevas* adicciones (juego, pantallas, redes sociales, *drogas* de diseño, de prescripción, etc.), los efectos adversos de los medicamentos, los derivados del cambio climático o, simplemente, la desinformación en aspectos de salud^6^.

No es sólo un problema de financiación. Los riesgos emergentes, y buena parte de los tradicionales, van a requerir en muchos casos nuevos o renovados abordajes, incluyendo estrategias transversales (salud en todas las políticas) o una mayor responsabilidad comunitaria e individual. También cambios radicales en los sistemas de información de salud pública (que no son sólo los de vigilancia epidemiológica de enfermedades transmisibles^7^) y en la *inteligencia* de salud pública, entendida como la capacidad de incorporar las evidencias científicas para identificar y anticipar riesgos y, también, las estrategias más efectivas para abordarlos

Una primera estrategia pasa por el desarrollo de renovados sistemas de información. Robustos, modernos, interoperables y seguros. Capaces de proporcionar datos fiables en tiempo-real sobre potenciales amenazas e identificar grupos de riesgo, para orientar una rápida toma de decisiones. Implica el enlace con otros sectores (incluyendo la atención sanitaria y sociosanitaria) y con otras jurisdicciones, reduciendo los intercambios fragmentados, la insuficiente granularidad de los datos (especialmente en determinantes socioeconómicos) y las demoras en su disponibilidad. Un sistema de información no es sólo la recopilación, uso y almacenamiento de datos, o las plataformas, el *software* (incluyendo la inteligencia artificial) y el *hardware* que les dan soporte. Incluye la inteligencia *natural* (los recursos humanos altamente competentes, incluida la investigación), para transformar los datos en información capaz de orientar la toma de decisiones.

Una segunda estrategia pasa por establecer y reforzar las alianzas y colaboraciones con las administraciones públicas de otros sectores y con otros agentes para mejorar, mantener y proteger la salud de las poblaciones. La coordinación con sectores no directamente sanitarios (vivienda/urbanismo, infraestructuras y transportes, servicios sociales, educación, trabajo, inmigración/interior, medio ambiente, economía, industria, ocio, alimentación, agricultura, hacienda...) es extraordinariamente compleja en organizaciones muy jerárquicas, con campos de acción definidos, cultura de *territorialidad* y cuyo único objetivo no es la salud. Pero son necesarias para abordar estrategias clave de salud pública, incluyendo actuaciones sobre la desinformación^8^.

Ambas actuaciones requieren reclutar, contratar, desarrollar, desplegar y retener un personal altamente competente, diverso y resistente^9^. Mejor formado y con mayores capacidades en las diferentes áreas que componen la salud pública, una disciplina híbrida donde las haya. Los recursos humanos de salud pública han ido disminuyendo. Envejecen. Y pesa el impacto de la pandemia pero también las cargas burocráticas, el clima laboral, la cultura organizativa administrativa y una carrera profesional más funcionarial que técnica. Adicionalmente, la incorporación de recursos viene lastrada por la ausencia de planificación, de actualización de perfiles laborales (no sólo salubristas, sino comunicación, ciencias de datos, competencias culturales y lingüísticas, …), y de una formación reglada adaptada a los perfiles y competencias necesarios en la salud pública del siglo XXI.

También importa la buena gestión pública que, con independencia del gobierno de turno, no es solo una cuestión de leyes y reglamentos. Requiere el rediseño de las estructuras, procesos, roles y responsabilidades del Estado y de las CCAA. No sólo en situaciones de emergencia, y no tanto para recentralizar como para mejorar la colaboración, la coordinación y la suficiencia en todas las jurisdicciones. También requiere desarrollar la *independencia* de las estructuras de salud pública. Aunque la salud pública tenga un enorme componente político, también tiene un extraordinario componente técnico y es importante distinguir las decisiones en ambos terrenos.

Hay avances en el espacio europeo (*EU4Health Programme*, *Europe’s Beating Cancer Plan,* transformación digital de los sistemas sanitarios, etc.). Más incierto parece el desarrollo de la Agencia Estatal de Salud Pública, pospuesto por la convocatoria electoral y con muchos interrogantes sobre contenidos, estructura, independencia, recursos humanos y presupuestos. Pasada la pandemia, con otros temas centrando la atención de la población y mientras va entrecerrándose la ventana de oportunidad, parece reinstalarse en nuestras administraciones sanitarias la acostumbrada escasa ambición en la transformación de la salud pública. Es un futuro posible. Entre otros futuros posibles que se van conformando en el día a día. Como decían algunas de las personas que participaron en la elaboración de la Ley de Salud Pública: “*Nuestra exigencia de que la ley se desarrolle y aplique debe plasmarse en el diálogo técnicamente riguroso, en la cooperación horizontal y vertical, y en acciones de abogacía en aquellos ámbitos de poder en que se dirime desarrollar la nueva norma o marginarla […] En buena medida, está en nuestra mano que en España la salud pública pase a ocupar un lugar más central en las políticas públicas y privadas, en el sistema económico y en los valores ciudadanos*”^10^.
